# Bridging the lesion—engineering a permissive substrate for nerve regeneration

**DOI:** 10.1093/rb/rbv012

**Published:** 2015-08-10

**Authors:** Liliana R. Pires, Ana P. Pêgo

**Affiliations:** ^1^INEB—Instituto de Engenharia Biomédica, Universidade do Porto, Rua do Campo Alegre 823, 4150-180 Porto, Portugal;; ^2^Instituto de Investigação e Inovação em Saúde, Universidade do Porto, Portugal;; ^3^Faculdade de Engenharia—Universidade do Porto (FEUP), Porto, Portugal and; ^4^Instituto de Ciências Biomédicas Abel Salazar (ICBAS), Universidade do Porto, Porto, Portugal

**Keywords:** Spinal cord, nerve regeneration, scaffolds, biomaterials, drug delivery

## Abstract

Biomaterial-based strategies to restore connectivity after lesion at the spinal cord are focused on bridging the lesion and providing an favourable substrate and a path for axonal re-growth. Following spinal cord injury (SCI) a hostile environment for neuronal cell growth is established by the activation of multiple inhibitory mechanisms that hamper regeneration to occur. Implantable scaffolds can provide mechanical support and physical guidance for axon re-growth and, at the same time, contribute to alleviate the hostile environment by the *in situ* delivery of therapeutic molecules and/or relevant cells. Basic research on SCI has been contributing with the description of inhibitory mechanisms for regeneration as well as identifying drugs/molecules that can target inhibition. This knowledge is the background for the development of combined strategies with biomaterials. Additionally, scaffold design is significantly evolving. From the early simple hollow conduits, scaffolds with complex architectures that can modulate cell fate are currently being tested. A number of promising pre-clinical studies combining scaffolds, cells, drugs and/or nucleic acids are reported in the open literature. Overall, it is considered that to address the multi-factorial inhibitory environment of a SCI, a multifaceted therapeutic approach is imperative. The progress in the identification of molecules that target inhibition after SCI and its combination with scaffolds and/or cells are described and discussed in this review.

5th China-Europe Symposium on Biomaterials in Regenerative Medicine (CESB 2015) Hangzhou, China April 7–10, 2015

## Spinal cord injury

Spinal cord injury (SCI) can be caused by compression, contusion, penetration or maceration of the spinal cord tissue, being very heterogeneous in cause, as well as in outcome. A lesion inflicted to the spinal cord leads to the interruption of motor and sensory neuronal pathways, resulting in the loss of motor, sensory and involuntary functions below the point of injury. Depending on the severity and location of trauma, SCI can be complete or incomplete, leading to different degrees of functional impairment. The primary injury triggers widespread cell death, including of neurons, oligodendrocytes, astrocytes or precursor cells. In parallel, oedema, ischemia and haemorrhage take place, resulting in the enlargement of the damaged area and creating, ultimately, a fluid-filled cyst. Subsequently, in the sub-acute phase of SCI, a cascade of events that constitute the secondary damage begins with oligodendrocyte apoptosis and loss of myelin, glutamate excitotoxicity, increase of free radicals and inflammation. This secondary injury results in a protracted period of tissue destruction. In the chronic phase, a glial scar is formed. Overall, the lesion site constitutes a particularly hostile scenery for axonal regeneration (see [1–4] for a review).

For a long time it was considered that neurons from the central nervous system (CNS) could not regenerate and the field of research on regeneration on the follow up of a SCI was quiescent. About three decades ago the first indications that regeneration can occur in the CNS were obtained using peripheral nerve grafts in the CNS [5, 6]. More recently, it was demonstrated that, although for short distances, axonal sprouting occurs after lesion, contributing to compensatory recovery and to the formation of new pathways that bypass the lesion [7]. Despite this regenerative potential, the fact is that after SCI the interrupted neuronal connections are not rewired and the impaired functions cannot be completely restored. This failure is mainly attributed to the establishment of an inhibitory environment for regeneration, namely by activation of inhibitory pathways, formation of a cavity (that withdraws the physical support for re-growth) and by the formation of a scar tissue that constitutes a real physical hurdle for regeneration [8–10]. Nevertheless, the last thirty years of research contributed with important findings, both at the molecular and cellular level, on the mechanisms underlying re-growth inhibition after SCI. Although these still did not succeed in being translated into the clinical setting, they formed solid ground for the current view on regenerative therapies to be applied in the aftermath of SCI, and the field agrees that to address such a multi-faceted inhibitory environment a combinatorial therapeutic strategy will be required [4].

## In the aftermath of SCI—the methylprednisolone debate

The current interventions in SCI are limited to spinal stabilization, rehabilitation and complication-prevention [11]. For some time, the recommended pharmacological treatment for SCI was the systemic administration of high doses of methylprednisolone (MP). MP is a synthetic glucocorticoid with anti-inflammatory and antioxidant properties, thought to induce neuroprotection and reduce the secondary damage upon injury [12]. A clinical trial in 1990 indicated the bolus injection of MP (30 mg/kg) during the first 8 h after injury as a mean to improve neurological recovery [13]. Based on this report, MP has been prescribed worldwide for non-penetrating acute SCI. However, the use of MP has been debated as well as the design of that clinical trial, and the performed data analysis was considered of dubious value [14, 15]. Other studies have reported limited beneficial effect of MP and secondary effects caused by the high dose administrated, like gastric bleeding [14, 16]. Additionally, a randomized clinical trial for head injury demonstrated that the mortality rate increases 2% with administration of MP [17]. Still, the controversy remains as there is recent experimental data supporting the use of MP for SCI [18–20] but also negative reports are being published [21, 22]. Consequently, the use of MP is no longer ‘standard of care’ for acute SCI, although it is still in medical practice.

## Inhibitory signals and therapeutic approaches

**Figure 1.**
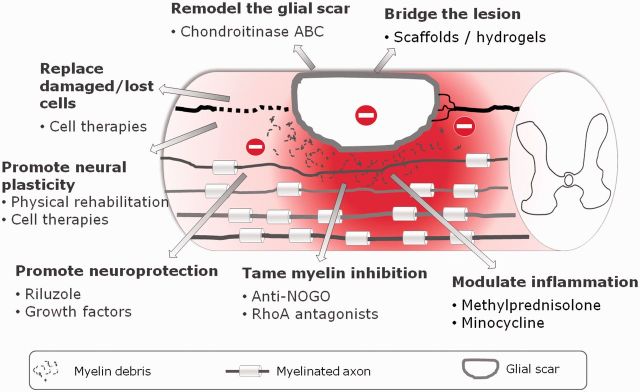
Spinal cord injury (SCI) therapeutic approaches currently under investigation.

Lesion to the spinal cord leads to the activation of a number of events that turn the lesion site into a very hostile substrate for axonal re-growth. Three processes have been identified as main contributors to the inhibitory environment: the activation of an inflammatory response, the accumulation of myelin debris and the formation of the glial scar. Based on the multiple sources of inhibitory signalling, diverse strategies have been proposed to address it. Overall, these strategies focus on (i) promoting neuroprotection of uninjured neurons, (ii) suppressing inhibitory signalling, (iii) replace damaged/death cells, (iv) induce neuroplasticity and/or (v) bridge the disrupted neuronal connections (see Fig. 1).

### Inflammation and immunomodulation

When a lesion occurs, mechanical forces contribute to the initial disruption of the blood brain barrier (BBB). The increase of the BBB permeability is taken as a prelude to the inflammatory response elicited by CNS trauma [23]. This triggers activation of endothelial and glial cells, promoting the release of vasoactive molecules—reactive oxygen species, kinins, nitric oxide and histamines—that influence endothelial function and enhance the BBB permeability, contributing to the enlargement of the injured area, exacerbation of damage and neurotoxicity. Moreover, inflammatory cells are recruited, namely: microglia (resident macrophages of the CNS), monocytes, neutrophils and lymphocytes. This process occurs within days after injury. Nonetheless, high levels of pro-inflammatory cytokines, such as interleukin (IL)-2 and IL-6, are detected in patients with chronic SCI, pointing to the existence of a continuous and prolonged inflammatory process [24].

Traditionally, inflammatory cell infiltration in the CNS is regarded as pathological [25]. However, the role of inflammation and inflammatory cells after SCI has been issue of active debate. Neuroinflammation is considered a dual-edged sword and both neurotoxic and neuroprotective properties are ascribed to inflammatory cells [16]. Macrophages were proposed to be the secondary damage effectors in SCI and their depletion showed to enhance axonal sprouting and improve motor function in a contusion SCI model [26]. On the other hand, injection of macrophages was explored as mean to improve recovery after SCI. M. Schwartz’s group defends that a well-controlled innate and adaptive immune response is pivotal for repair in SCI [27, 28], basing their claim on the observation that the injection of ‘alternatively *ex vivo* activated macrophages’ in a spinal cord lesion promotes functional recovery [29]. Macrophages activated prior injection in the spinal cord by co-culturing with peripheral nerves showed increased phagocytic and proteolytic activity, and reduced pro-inflammatory bias. In the late nineties, this work was very controversial. Nowadays, macrophage polarization is well accepted (see [30–32] for review) and to learn how to control the opposing functions that these cells can exert depending on their phenotype is a topic of interest in many different research fields, including in SCI [28, 32, 33]. In fact, the modulation of macrophage function is the rationale behind the use of some neuroprotective drugs, such as MP, minocycline [34] or other anti-inflammatory molecules, like IL-10 [35, 36]. MP is the only drug that achieved the clinical setting for SCI treatment as described previously in this review. Minocycline is an antibiotic with anti-inflammatory properties. The drug is known due to its immunomodulatory properties, being able to tune the expression of cytokines, attenuate oligodendrocyte and microglia cell death, and improve functional recovery in rat SCI models [37, 38]. In the phase I/II clinical trial for acute SCI, minocycline showed to be safe and, although the functional evaluation did not accomplish statistical significance, there is a clear tendency towards improvement that encouraged the phase III clinical trial [39], currently recruiting participants [40].

### Taming myelin inhibition

Damage to the spinal cord results in neural cell death. In particular oligodendrocyte cell death leads to axon demyelinization and can result in neuron degeneration. Additionally, the loss of contact between oligodendrocytes and axons, due to neuron cell death, can also induce oligodendrocyte degeneration or even death.

It is well known that oligodendrocytes and myelin are inhibitory substrates for axonal growth [41, 42] and myelin debris accumulation at the injury site is one of the critical events halting regeneration. Several molecules present in myelin were already identified as inhibitors for axonal growth including Nogo-A, myelin-associated glycoprotein, oligodendrocyte-myelin glycoprotein and its downstream effectors such as Nogo-66 receptor [43], Ras homolog gene family member A (RhoA) and Rho-associated protein kinase (ROCK) [44, 45].

The inefficient clearance of myelin debris by microglia and macrophages in the CNS (in opposition to the effective cleaning mediated by macrophages in the peripheral nervous system where regeneration occurs [46]) is considered, by several authors, as the main responsible for CNS residual ability to regenerate [1]. In view of that, the injection of autologous macrophages at the injury site has been investigated in a clinical trial (ProCord, Proneuron Biotechnologies). Improvements were detected in 5 out of the 16 acute phase patients [25], but when tested in phase II, the treatment showed no improvement on the primary outcome comparing to non-treated individuals [47].

Alternatively, blocking myelin-mediated inhibition was explored with the use of antibodies against Nogo-A. Three different antibodies have been tested in pre-clinical models over the last 15 years [48–50]. A Phase I clinical trial using an humanized anti-Nogo antibody, ATI355 produced by Novartis, showed the safety of the treatment, but results related to the efficacy were not disclosed [51]. The anti-Nogo therapy is applied in acute phase patients, since the time window for application of this therapy is limited, showing a progressive loss of responsiveness [52].

Inactivation of RhoA, a downstream effector of myelin-associated pathways, has been shown by several groups to overcome axonal growth inhibition by individual inhibitors and by myelin in general. Inactivation of RhoA by the application at the site of injury of the toxin C3 (*Clostridium botulinum*) promotes an extensive regeneration and functional recovery in mice [53]. Hindlimb recovery was also reported after administration of the toxin or Y27632—a specific inhibitor for ROCK [44]. RhoA is a convergence molecule for many inhibitors of axonal regeneration and it is, for that reason, a promising target for SCI therapeutics [25]. The first results of a phase I clinical trial using a cell-permeable Rho antagonist, called BA-210 (Cethrin, a recombinant protein), were published by Alseres Pharmaceuticals. Cethrin was administered by extradural application with a fibrin sealant to patients with acute cervical SCI during spinal decompression surgery conducted within 72 h after injury [54]. Twelve months after intervention, 5 out of 13 patients (38%) showed marked recovery of motor and sensory function after treatment, as measured by a 2-grade improvement or higher in the American Spinal Cord Injury Association (ASIA) impairment scale [55]. A phase IIb trial was planned, sponsored by Bioaxone Biosciences, but by the end of 2014 it was withdraw, because the drug was licensed to Vertex Pharmaceuticals.

Ibuprofen is used worldwide as a non-steroidal anti-inflammatory drug. Its action has been attributed to the inhibitory effect on cyclooxygenase and it is used to reduce pain, fever and acute inflammatory reaction [56, 57]. In 2007, it was described for the first time that ibuprofen can inhibit the activation of RhoA in a SCI scenario [58]. The drug prevents myelin inhibition of neurite outgrowth by reducing RhoA activation *in vitro*, and when administrated *in vivo* after SCI improves functional recovery [58, 59] and corticospinal axonal regeneration [58]. Even though a recent re-assessment study reports only partial replication of the results obtained in 2007 [60], the number of publications that demonstrate positive effects of ibuprofen on nerve regeneration is significant [61, 62] and the use of this drug is considered very promising [63]. Indeed, a clinical trial to assess safety of the administration of the drug in SCI patients was launched recently and is recruiting patients [40].

### Overcoming the glial scar

As a lesion to the spinal cord progresses into a chronic phase, a glial scar is formed. The glial scar is mainly an astrocytic tissue consisting of hyperfilamentous astrocytes, with processes tightly packed, many gap and tight junctions and limited extracellular space [64]. The scar is formed to isolate the injury, reseal the BBB and prevent the damage of the spared tissue and the spreading of excitotoxicity and cytotoxic molecules [65]. However, the glial scar represents a mechanical barrier for axonal re-growth, being also a source of chemical inhibitors for regeneration. Chondroitin sulphate proteoglycans (CSPGs) produced by astrocytes are present in the extracellular matrix in the CNS and, in pathological conditions, are the major constituent of the glial scar, being an inhibitory signal for axonal growth [66]. In this context, CSPGs have been targeted in SCI therapeutics and it was demonstrated that digestion of CSPGs by chondroitinase ABC promotes axon regeneration and plasticity, leading to functional recovery of locomotor and proprioceptive behaviour after SCI [67]. Treatment with this enzyme is likely to be advantageous even 7 days after injury [68], making this strategy particularly interesting for non-acute spinal cord lesions. However, the origin of the enzyme (bacteria), as well as the degradation products formed, have been issue of concern due to the possibility of triggering a specific immune response, hindering clinical translation [69]. Moreover, these degradation products can exert some inhibitory influence on the growth of spinal axons [70]. The use of lentivirus-based delivery of a modified chondroitinase gene (that encodes for a secreted form of the enzyme that can be expressed by mammalian cells) is under investigation, as a mean to circumvent some of these caveats [71, 72].

### Protecting injured neurons and repopulating the injury

Neurotrophins are molecules with interest in the context of SCI due to their important role in neural development, survival and regeneration [73]. Injection of nerve growth factor (NGF) [74], brain-derived growth factor (BDNF) or neurotrophin-3 (NT-3) [75] was performed in SCI animal models with different degrees of success. Bradbury *et al.* [75] found that NT-3 is significantly more effective than BDNF promoting the growth of injured axons in a rat dorsal crush model. A large-scale animal study indicated that the local application of BDNF can induce neuroprotection if applied at high doses and shortly after trauma [76]. Other neurotrophic factors such as glial-derived growth factor (GDNF) [75] and insulin-like growth factor (IGF-1) [76] were already proposed to treat SCI. Regardless the promising results obtained *in vitro* and in animal models, a clinical trial using systemic delivery of growth factors for diabetic neuropathy showed limited efficacy and significant side effects [77], slowing down the progress of new clinical studies with these molecules. Currently, the use of neurotrophic factors appears to be particularly relevant when combined with drug/gene delivery strategies and/or cell-based therapies [4].

Other molecules have been investigated for treatment of SCI based on their neuroprotective effects, being riluzole the most successful example. Riluzole is a sodium channel blocker and the rationale for its use in acute SCI is that removing sodium excess upon injury, neuronal depolarization is prevented, reducing the accumulation of glutamate and excitotoxicity. It has been shown that the administration of riluzole after SCI in rats reduces oedema and improves motor recovery [78]. A clinical trial enrolling 36 patients was carried out, aiming at evaluating the safety of the drug administration within 12 h after injury. Full results await publication, but a phase II/III trial is currently recruiting [40].

According to clinicaltrials.gov [40], ∼10% of the open clinical trials for SCI use cellular therapies. The implantation of cells (including stem cells of different origin) in SCI holds the promise of repopulating the injury site, promoting the production of growth factors and cell plasticity. Current literature suggests that cell-based therapies will be of particular interest in acute or sub-acute phases of SCI, since transplantation in chronic patients showed to yield limited functional benefit [79]. The use of cells for SCI treatment was reviewed in detail elsewhere [79–83].

## Biomaterial-based strategies for SCI—Engineering an implantable bridge

**Figure 2.**
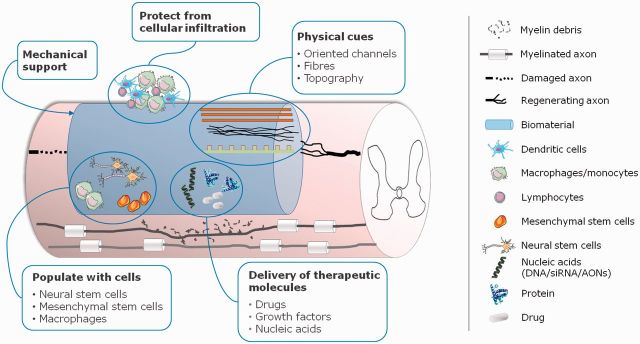
Biomaterials-based approaches to spinal cord injury (SCI) therapeutic management. siRNA, small interference RNA; AONs, antisense oligonucleotides.

Designing a bridge to re-connect the injured spinal cord encloses several challenges, namely facing the hostile environment at the lesion site or providing adequate mechanical support. Nonetheless, to develop an implantable device provides a unique opportunity to combine different therapeutic signals in a single platform (Fig. 2). Nerve regeneration research based on the use of biomaterials was primarily focused on the development of scaffolds that connect the lesion site, filling the cavity formed upon lesion, providing physical support and a path for axonal re-growth and limiting cellular infiltration from the periphery. These scaffolds have been evolving from the simple hollow conduit to more sophisticated devices with improved physical guidance architectures combined with cells, drugs or nucleic acids material. Overall, scaffolds are expected to allow the modification of the inhibitory environment at the lesion site and, consequently, contribute to the process of regeneration [84]. This review focused in particular on the evolution of nerve conduits and porous scaffolds in terms of design and as versatile platforms for the development of combinatorial therapeutic strategies.

### Candidate biomaterials

The use of nerve conduits to bridge a nerve lesion was firstly explored for peripheral nervous system regeneration, as an alternative to autologous nerve grafting (see [85, 86] for a review). Upon the evidence that neurons from the CNS can regenerate into peripheral nerve grafts [5], the development of nerve conduits was extrapolated to the treatment of SCIs. Ideal requisites of such nerve conduits include permeability to oxygen and nutrients, limited swelling, flexibility without kinking [85], versatility in terms of architectures and dimensions, should allow sterilization as well as to be easy to handle and suture [46].

Several materials have been used to prepare bridges for SCI, in particular biodegradable materials, either natural or synthetic, as reviewed in Table 1. The use of synthetic materials to prepare nerve conduits has the advantage of the control over manufacturing parameters and the possibility to fine-tune the chemical structure, mechanical properties and degradation rate (when applicable). Among them, aliphatic polyesters, like poly(lactide) (PLA), poly(glycolide) (PGA) and their copolymers, have been the most explored [87–89], probably encouraged by the fact that these are approved by the Food and Drug Administration (FDA) (see Table 1). Another popular synthetic polymer in the nerve conduit research field is poly(ε-caprolactone) (PCL) [90, 91], despite the limited number of *in vivo* studies concerning its application in a SCI scenario. PCL has a slower degradation rate in comparison to PGA or PLA, and to tune its properties, it has been co-polymerized with 1,3-trimethylene carbonate [92, 93] or ethyl ethylene phosphate [94]. Porous scaffolds of poly(trimethylene carbonate-co-ε-caprolactone) [P(TMC-CL)] showed to support peripheral nerve regeneration *in vivo* [95]. In the CNS context, P(TMC-CL) showed to stimulate neuronal polarization and axonal elongation, favouring neurite outgrowth even when in an inhibitory environment [96].

**Table 1.** Materials studied for nerve regeneration and tested in SCI models

Polymer	Nature	Type of bridge	Combination therapy	Ref.
PLA	Synthetic, degradable	Single walled conduit, electrospun fibres	Drug release	[109–111]
PLGA	Synthetic, degradable	Multiple channel; electrospun fibres	Plasmid DNA; Schwann cells; self-assembling peptides for growth factor delivery; drug delivery	[112–116]
PCL	Synthetic, degradable	Porous scaffold	Neural stem cells	[117, 118]
PCLEEP	Synthetic; degradable	Electrospun fibres	Drug delivery, siRNA	[94, 119, 120]
P(HEMA) and copolymers	Synthetic; non-degradable	Hydrogel; scaffold	Drug delivery; modified with SIKVAV	[121–124]
Chitosan	Natural; degradable	Porous scaffold, conduit	Endothelial cells; collagen hydrogel as filler; bone marrow stem cells	[98, 99, 125–127]
Collagen	Natural, degradable	Electrospun fibres; oriented pore channels; hydrogel	Growth factors; chondroitinase ABC, stem cells	[100, 125, 128–130]
Gelatin	Natural; degradable	Scaffold	Neuronal + endothelial cell lines	[131, 132]
Agarose	Natural	Hydrogel/scaffold	Growth factors	[20, 133]
Hyaluronic acid	Natural	Porous scaffold, scaffold coating, hydrogel	Growth factors, cells	[134–137]

PLA, poly(lactide)*;* PLGA, poly(lactide-co-glycolide); PCL, poly(ε-caprolactone); PCLEEP, poly(ε-caprolactone-co-ethyl ethylene phosphate); P(HEMA), poly(2-hydroxyehtyl methacrylate); siRNA, small interference RNA.

Natural polymers can be generally considered as biocompatible and non-toxic. These properties have made natural polymers attractive materials for the preparation of nerve tissue engineering constructs [97]. Chitosan [98, 99] and collagen [100] are popular natural materials used to prepare nerve conduits (for the peripheral or CNS). Other materials applied as scaffolds for SCI include hyaluronic acid, agarose or gelatin, (see Table 1). To combine the advantages of both natural and synthetic polymers, blending have been actively investigated, as shown by the different combinations of PCL with gelatin [101], collagen [102] or chitosan [103].

To enhance the bioactivity of conduits, these have been modified with cell adhesion peptides [104, 105] or extracellular matrix proteins like fibronectin [88, 99, 106, 107], or laminin [88, 89, 98, 108]. The incorporation of extracellular components on nerve bridges holds the premise of improving cell adhesion and promoting axon path finding.

Some of the above mentioned materials could be processed as solid conduits as well as injectable materials. The use of injectable materials is considered of particular interest in SCI lesions where the dura matter is not compromised. In this case, the use of *in situ* crosslinking hydrogels that can fill the cavity formed without causing further damage to the tissue is preferable to the implantation of a solid device [84]. Detailed revision on hydrogel-based strategies for SCI is out of the scope of this review and can be found elsewhere [82, 138].

An important class of materials that has also been investigated for the preparation of nerve conduits is the one of conductive polymers. It has been shown that electrical stimulation increases neurite outgrowth [139]; therefore, to provide scaffolds with conductive properties can positively contribute for the regeneration process. However, synthetic conductive polymers, like poly(pyrrole) and poly(aniline), have poor biocompatibility, biodegradability and are difficult to process in different scaffold designs. In view of the above, these polymers have been applied as coating or blends with other synthetic [140, 141] or natural [142] polymers as a mean to improve the construct biocompatibility while maintaining the electrical conductance and, ultimately allowing the stimulation of neurite outgrowth.

### Scaffold design—architecture and topography

It is well accepted that axonal growth is useless if axons wander randomly and that axons need guidance for functional regeneration to occur [143]. However, the use of hollow conduits to bridge the lesion area in the spinal cord showed limited success [98, 109, 125]. Oudega *et al.* [109] implanted a PLA single walled conduit in a complete transection SCI model and showed that the tube collapsed soon after implantation, compromising axonal regeneration. Conduits made of chitosan [125] or chitosan modified by plasma treatment with laminin [98] were implanted in the spinal cord in a 4 mm gap resection model, but no improvement on axonal re-growth or function was detected comparing to injured rats that did not received a conduit. Furthermore, there is mounting evidence that axons orient their processes according to the substrate and are sensible to patterns on the surface the processes contact with [144–147], suggesting that to integrate these features in the design of nerve conduits can improve the guidance performance of these devices. In view of the above, the development of an internal lumen for such conduits is being investigated, namely the incorporation of smaller channels, nanofibres or hydrogels. These can favour cell attachment due to a higher surface area and can also serve for the incorporation of other molecular cues, like extracellular matrix components and growth factors [148].

A very elegant work has been conducted by Shea and collaborators using multiple channel poly(lactide-co-glycolide) (PLGA) bridges in the spinal cord. The scaffolds containing channels between 150-250 μm were prepared by gas foaming and particulate leaching and showed to maintain their integrity 13 days after subcutaneous implantation [149]. When implanted in the spinal cord, limited macrophage infiltration was observed and the conduits were stable at least for 6 weeks [112]. Complete degradation of the scaffolds was observed 6 months after implantation in the spinal cord [150]. Furthermore, they demonstrated that the preparation of these conduits is compatible with the delivery of bioactive growth factors [149, 151, 152], DNA [113, 151] or lentivirus encoding for growth factors [153, 154], showing significant improvements in regeneration in SCI models.

More complex internal lumen architectures can be achieved using computer aided mould design and solid freeform fabrication [155]. Using this approach, seven channel conduits were prepared by injection moulding of a number of synthetic polymers. After implantation in a complete transection model for SCI combined with Schwann cells, conduits of an oligo(polyethylene glycol) fumarate, positively charged, hydrogel showed better results in terms of number of axons colonizing the scaffold comparing to PLGA scaffolds [114, 155]. Nevertheless, none of the materials tested achieved a functional improvement 4 weeks post-implantation in a SCI model [114].

Although still to be tested *in vivo*, collagen multichannel conduits based on electrospun fibres are being investigated and have shown great potential as nerve guides due to the very high surface area of the structure, mimicking the fascicles in nerve [156]. The preparation of scaffolds with oriented microchannels has also been explored using freeze-drying technique for polymers like collagen [129] or gelatin [131] and blends with chitosan [157].

Alternatively to channels, hollow conduits have been filled with hydrogels [100, 158]. In the context of SCI, a large-scale study was performed using a chitosan conduit filled with a semi-fluid collagen reports a significant improvement on the number of axons that can cross the bridge in rats where collagen was used to fill the chitosan tube, leading also to a significant increment in the Basso, Beattie and Bresnahan locomotor rating scale [125].

Electrospun scaffolds have been widely investigated for peripheral nerve regeneration [87, 89, 94, 101, 119, 159–161], and more recently, also explored in the context of SCI [100, 110, 115, 116, 162–165]. The first report testing electrospun fibres in a SCI scenario was published in 2007. Meiners *et al.* [162] implanted a fabric based on randomly oriented polyamide fibres in a hemisection SCI model. The results were unsatisfactory, since the fabric tended to fold, impairing axonal outgrowth. This study highlighted the importance of developing oriented nanofibrillar scaffolds for directing axonal growth. Indeed, it has been shown that neurite extension is increased when cells are cultured on aligned fibres, comparing to random substrates [161, 166]. Hurtado *et al.* tested PLA conduits consisting of a rolled mats of random or aligned micrometer fibres implanted after complete transection of the spinal cord. Although the functional recovery was not assessed in the study, the authors showed a robust axonal regeneration in conduits with aligned fibres, 4 weeks after implantation [110]. Conversely, a preliminary *in vivo* study using collagen nanofibrous scaffolds showed limited success after 4 weeks of implantation [100]. The limited results are believed to be related to the degradation of the conduit and the size of the fibres. Fibres with a mean diameter around 200 nm [100] seem to be less effective guiding axonal growth than micrometer fibres used in other studies [110], although the role of fibre diameter on axonal growth after SCI needs to be investigated in more detail [100].

An alternative scaffold design using electrospun fibres was proposed by Gelain *et al.* [115]. The authors implanted 4 weeks after the infliction of a contusive lesion layers of electrospun tubes with 210 μm diameter filled with self-assembling peptides and fixed by a PLGA / PCL electrospun sealing lamina. To implant the scaffold, the scar tissue and debris were removed and then the cavity was filled. The results showed significant axonal growth inside and between the channels spanning the lesion. Functional motor recovery was also observed, being statistical significance achieved only 22 weeks after scaffold implantation [115].

The evidence shows that not only conduits and oriented channels can guide axonal growth; topographic cues also play a major role driving cell growth and conditioning their behaviour. Research on the effect of topographic cues, and in particular of aligned substrates, have been primarily focused on neurons. Aligned fibres and micropatterned surfaces showed to promote directional axonal growth [90, 91, 100, 167]. Moreover, it has been demonstrated that aligned substrates affect adult stem cell differentiation, potentiating differentiation into the neuronal lineage [168]. However, for functional regeneration to occur, not only neuronal oriented growth is required. The lack of organization of glial cells after injury is also associated to regeneration failure. In fact, regeneration through an aligned fibrous scaffold was found to be supported by astrocyte migration along the fibre direction [110]. Indeed, astrocytes orient their filamentous structure according to the topography of the surface [107, 163, 169, 170]. Moreover, it has been reported that the contact of astrocytes with fibres is able to promote a decrease on glial fibrillary acidic protein expression [170] and an increase in glutamate uptake, what can contribute for neuroprotection *in vivo* [107]. Furthermore, controlled micropatterned scaffolds showed to influence astrocyte calcium signalling [171], being capable of reverting mature astrocytes into radial glia-like cells, and consequently to a more pro-regenerative phenotype [172]. Similarly, PLA aligned fibres were found to favour cell de-differentiation towards radial glia or neuronal progenitors. This positive effect was attributed to the physical cues provided by the fibres, but also to the release of lactate due to fibre biodegradation [173]. Published research shows that cells from the immune system can also respond to topographic cues and this may have a critical impact on the regeneration process. It has been obeserved that macrophages cultured on electrospun fibres of PLA [174] or PCL [175] secrete less pro-inflammatory cytokines comparing to cells cultured on flat solvent-cast films, suggesting improved biocompatibility of these surfaces [174]. McWhorter *et al.*[176] proposed the use of surface topography to modulate macrophage phenotype by controlling cell shape. To translate this knowledge into nerve conduits can be a relevant approach towards spinal cord regeneration taking in consideration macrophage role on secondary injury after SCI [26]. Although microglia is responsible for the early pro-inflammatory environment after SCI [177], little is known about the response of these cells to surface topography. The effect of nanostructured silicone on BV-2 (a microglia cell line) cell adhesion was investigated, showing that cells can undergo marked morphogenic changes, according to feature size (30 nm–2 µm) [178]. Microglia can also interact mechanically with nanostructured 3D features, like micro-pillars (4.7 µm of height), adapting actin cytoskeleton to these structures [179]. This microglia morphologic plasticity was also described by us for primary cultures seeded on P(TMC-CL) substrates. On fibrous surfaces microglia showed small cytoplasm and more ramified structure, comparing to flat solvent-cast films [180]. However, in our study we did not observe a correlation between the morphologic alterations and the cytokine release profile, in opposition to previous reports by others using macrophages [174, 175]. Still, the topography of the surface showed to influence microglia ability to form multinuclear giant cells, when cells are challenged with myelin in the culture media [180]. These results highlighted the need to understand the functional relevance of the reported alterations in cell morphology. Furthermore, no extrapolations from the macrophage behaviour can be done, as microglia react differently to stimuli, despite sharing relevant lineage features with macrophages [181–183].

### Drug releasing scaffolds

The localized delivery of the drugs/therapeutics described in the first part of this review to the spinal cord injured area has been explored as a mean to reduce side effects associated with systemic administration as well as a way to improve drug efficiency [20].

Drug delivery into the intrathecal space after lesion in the spinal cord have been explored using injectable hydrogels as carriers. Hydrogels based on hyaluronan and methyl cellulose were proposed for the delivery of neurotrophic factors [123, 136]. Similarly, injection of fibrin loaded with NT-3 showed to promote functional improvements after SCI, comparing to unloaded hydrogels [184]. Chondroitinase ABC has also been delivered through fibrin hydrogels to an injured spinal cord [185].

Alternatively, different drugs have been loaded in scaffolds to be implanted in the lesion and release therapeutic molecules *in situ*. Research on PLGA multiple lumen conduits showed that these scaffolds are compatible with the delivery of NGF [149], vascular endothelial growth factor or fibroblast growth factor [152], showing positive results when implanted in SCI model.

A number of researchers have focused on combining drugs with electrospun scaffolds. Electrospun fibres have been loaded with growth factors [94, 128] or cAMP [186], aiming to promote neuroprotection. An alternative approach was explored by combining 6-aminonicotinamide in PLA fibres as a mean to limit astrocyte proliferation [111], or loading ibuprofen in P(TMC-CL) electrospun fibres in order to reduce local inflammatory response [187]. Collagen fibres were also prepared for the delivery of NT-3 and chondroitinase ABC [128]. An option for nanofibrous scaffolds is to be applied as patches for drug delivery in the spinal cord. Fibrous patches loaded with rolipram, a small molecule that can enhance cAMP activity in neurons, and suppress inflammatory response, favouring nerve regeneration [188], showed to improve locomotor function in rats with SCI in comparison to unloaded patches from the third week on after implantation [116]. To better control the release of the drug and increase drug loading, the electrospun fibres were combined with an alginate hydrogel. However, the use of high drug doses showed to lead to toxic effects and an increased mortality rate [165].

Drug loading in nanoparticles subsequently delivered to the spinal cord within a scaffold has been investigated for drugs, such as MP or minocycline. An agarose hydrogel containing PLGA nanoparticles loaded with MP was implanted in a contusion model of SCI leading to reduced lesion volume and macrophage infiltration [20]. For the delivery of minocycline, drug loaded nanoparticles based on PCL and polyethylene glycol (PEG) were applied, being able to reduce activation and proliferation of microglia/macrophages *in vivo* [189].

Scaffolds containing genetic material can serve as depots for the *in situ* delivery of genes to cells at a lesion, potentially inducing the expression of a therapeutic protein for longer periods and higher concentrations, as compared with direct protein delivery [190]. In the context of nerve regeneration, PLGA disks loaded with poly(ethylene imine)-DNA nanoparticles containing a plasmid encoding for NGF showed to promote axonal elongation in dorsal root ganglia neurons co-cultured with human embryonic kidney 293T cells [191]. Particularly in a SCI scenario, lipid-DNA particles were incorporated in a PLGA channel bridge and a high expression of the reporter gene was detected in the spinal cord during 3 weeks [88]. However, to achieve functional improvements the implantation of conduits containing more efficient gene delivery vectors, namely viral vectors (lentivirus), was needed [153, 154]. A very promising alternative currently under investigation is to load scaffold with nanoparticles carrying small interference RNA (siRNA) [192, 193] or microRNAs [194] targeting inhibitory pathways of the regenerative process.

### Cellular bridges

Research in biomaterials to assist the delivery of cells to the CNS, and to the spinal cord has been primarily devoted to the use of hydrogels [82, 83, 195, 196]. Hydrogels appeared to address the need to improve cell survival and/or to modulate cell differentiation after implantation [197]. However, recent reports in the open literature demonstrate that the implantation of scaffolds colonized with cells represents a promising complement for design of effective cellular therapies. This strategy is the focus of this section.

Xue *et al.* [198] investigated the implantation of a chitin tube loaded with mesenchymal stem cells (MSC) immediately after spinal cord hemisection. The authors showed that the implantation of the scaffold was able to reduce scar formation, establishing a favourable environment for cell survival and proliferation. In a more complex approach, Zeng *et al.* [132] applied gelatin porous sponges populated with MSC manipulated *in vitro*. First, the cells were genetically engineered in order to induce overexpression of a NT-3 receptor, the tyrosine kinase C. Subsequently, the MSC were co-cultured with Schwann cells overexpressing NT-3. The authors demonstrated that this process favours MSC differentiation towards the neuronal lineage. The implantation of the scaffolds colonized with the engineered MSC after complete transection of the spinal cord showed to improve functional recovery comparing to plain sponges and to support integration in the host neural network [132].

A popular cell type applied in cellular therapies for SCI are neural stem cells (NSC), which can be obtained from different sources. NSCs are committed to the neural lineage and are believed to contribute to neuronal regeneration by favouring host axonal regeneration due to trophic effect and by replacing damaged/lost cells. PLGA multichannel conduits [199] and chitosan tubes [127] were implanted in the spinal cord combined with NSC, with some degree of success. Improvements on transplanted cell survival were reported, but no significant functional recovery was reported. A recent study describes the use of NSC obtained from induced embryonic fibroblasts seeded on gelatin-electrospun PLGA/PEG scaffolds. The scaffolds were prepared by assembling a gelatin sponge and a PLGA/PEG electrospun mesh and rolling it together into a cylinder. Eight weeks after implantation in a complete transected spinal cord, the scaffolds showed to reduce cavity formation, supporting NSC survival and proliferation. The authors suggested that cell survival contributed to functional recovery, however the study did not disclose the effect of the scaffold on itself, as scaffolds without cells were not tested [200]. Collagen porous sponges showed also to support NSC delivery and survival, being able to reduce the formation of a fluid-filled cyst comparing to non-treated animals when implanted in a 5 mm full resection gap model of SCI [130].

A different approach was explored by Jian *et al.* [201] that used a glioma cell line as extracellular matrix depositors to coat a chitosan tubular scaffold prior implantation. Interestingly, such enriched scaffolds showed to favour stem cell differentiation into the neuronal lineage *in vitro*. However, *in vivo*, modest improvements in function were found only if scaffolds were combined with a glycogen synthetase inhibitor, known to play a role on stem cell differentiation [201].

## Future perspectives

Much research in the field of SCI aims at the understanding of the fundamental cellular and molecular mechanisms that underlie this multi-factorial clinical condition. Immense progress has been made in the last decades and at present the field believes that regeneration can occur, at least to a certain extent, if the environment at the lesion site is tuned to a pro-regenerative context. Still, the major challenge is to develop effective therapies based on the advances achieved on basic research. As in other field of tissue engineering and regenerative medicine, biomaterials and scaffold structures are expected to play a key role on the design of such advanced therapies.

Nerve conduits have been investigated for long in the field of SCI, although their use in the clinical setting was never achieved. The design of these scaffolds has been increasing in complexity, as it is happening with the proposed therapeutic approaches. The development of combinatorial strategies deals with the difficult task of identifying the appropriate scaffold design, the best cell source and the correct drug/molecule cocktail and dose. This will be needed to respond to the multi-factorial inhibitory environment formed in the aftermath of a SCI. But important progress was attained recently, with the start of a clinical trial to assess the safety of the implantation of a PLGA/poly(lysine) scaffold in SCI patients [40]. Additionally, a more ambitious trial was recently launched in China, proposing to evaluate functional improvements after implantation of a collagen scaffold loaded with MSCs and growth factors [40]. To perform clinical trials with implantable scaffolds represents a major achievement that will certainly push forward the biomaterials and tissue engineering research in SCI. The expected data will be paramount for the establishment of such ‘bridges’ as powerful tools in the design of much awaited effective therapeutical strategies.
